# Enhanced thermal stability of lichenase from *Bacillus subtilis* 168 by SpyTag/SpyCatcher-mediated spontaneous cyclization

**DOI:** 10.1186/s13068-016-0490-5

**Published:** 2016-03-31

**Authors:** Jindan Wang, Yilin Wang, Xinzhe Wang, Dandan Zhang, Shuyu Wu, Guangya Zhang

**Affiliations:** Department of Bioengineering and Biotechnology, Huaqiao University, Xiamen, 361021 Fujian China; Biochip Laboratory, Yantai Yuhuangding Hospital Affiliated to Medical College of Qingdao University, Yantai, 264000 Shandong China

**Keywords:** SpyTag/SpyCatcher, Cyclization, Non-chromatographic purification, Lichenase, Thermal stability

## Abstract

**Background:**

SpyTag is a peptide that can form an irreversible covalent linkage to its 12 kDa partner SpyCatcher via a spontaneous isopeptide bond. Herein, we fused SpyTag at the N-terminal of lichenase and SpyCatcher at C-terminal so that the termini of lichenase were locked together by the covalent interaction between the partners. In addition, an elastin-like polypeptides tag was subsequently attached to the C-terminus of SpyCatcher, thereby facilitating the non-chromatographic purification of cyclized lichenase.

**Results:**

The study showed that the optimum temperature of the cyclized lichenase was about 5 °C higher in comparison to its linear counterpart. Moreover, nearly 80 % of the cyclized lichenase activities were retained after 100 °C exposure, whereas the linear form lost almost all of its activities. Therefore, the cyclized variant displayed a significantly higher thermal stability as temperature elevated and was resistant to hyperthermal denaturation. Besides, the Km value of the cyclized lichenase (7.58 ± 0.92 mg/mL) was approximately 1.7-fold lower than that of the linear one (12.96 ± 1.93 mg/mL), indicating a higher affinity with substrates.

**Conclusions:**

This new SpyTag/SpyCatcher cyclization strategy is deemed as a generalized reference for enhancing enzyme stability and can be effectively customized to the cyclization of various enzymes, hence a tremendous potential for successful application in the biocatalytic conversion of biomass to produce fuels and chemicals.

## Background

β-1,3;1,4-glucans are the main constituent of the cell wall of the endosperm of barely grains (i.e., oats and barley). β-1,3;1,4-glucans and lichenan are polysaccharides, which can be processed into value-added products and biofuels (i.e., ethanol). Besides, it shows great potential as renewable polymers. Lichenase (E.C. 3.2.1.73) has received notable attention, thanks to its significant role in exploring the feasibility of biofuel production [1], though some thermostable lichenases from various origins have been isolated, for example: *Bacillus* sp. UEB-S [2], *Aspergillus niger* US368 [3] and *Clostridium thermocellum* [4]. However, current commercially available lichenases may not be ideally suitable for biomass biocatalytic conversion because of its limited thermal stability. Thus, thermal stability modification for lichenase as well as other industrial enzymes, coupled with isolation of thermostable lichenases, has become a research focus during the past few decades [5, 6]. Indeed, conventional methods represented by directed evolution, and rational design have contributed to remarkable successes in improving the thermal stability of enzymes [7–10]. Nonetheless, inferior success frequencies for rational design, as well as time consuming in nature, have limited a broad utilization of these methods [11]. Therefore, it is desirable to develop a more efficient and simplified approach to improving the enzyme thermal stability. On the other hand, covalent cyclization of protein backbone can stabilize enzymes by reducing not only the conformational entropy of folded domains but also that of unfolded regions in cyclized enzymes, with most of their catalytic activities and functions retained [12–14]. There upon, enzyme cyclization was regarded as a novel efficient technique for improving its thermal stability and extensively applied in industrial biocatalysis. Current cyclization technologies include various strategies, namely, chemical synthesis combined with chemical ligation [15], intein-mediated protein trans-splicing [16, 17], protease-catalyzed traspeptidation [18], and genetic reshuffling [19]. However, there are still several drawbacks existing on the following fronts: (a) chemical ligation or cross-linking generally requires harsh reaction conditions that may disrupt enzyme conformations; (b) chemical synthesis method is more suitable for small molecule rather than macromolecular enzymes; (c) intein-mediated protein trans-splicing and protease-catalyzed traspeptidation sometimes result in molecule misfolding, a low expression of target protein, or generation of inclusion body.

Originally identified in *Streptcoccus pyogenes*, SpyTag spontaneously and specifically formed an intermolecular amide bond with its partner SpyCatcher. The reaction could proceed in a short time, with high yielding and rapid reconstitution, and the formation of amide bond is robust to a wide range of pH values, temperatures, buffer compositions, or in the presence of detergents. Therefore, SpyTag/SpyCatcher is deemed a novel molecular adhesion that plays a vital role in enzyme thermal stability improvement [20, 21]. Schoene and coworkers found that an increase in aggregation temperature (more than 60 °C) for β-lactamase was achieved using SpyTag/SpyCather sandwiching [22]. Besides, the catalytic activity of the cyclized β-lactamase was retained upon thermal stress. It is conceivable that SpyTag/SpyCatcher-mediated cyclization method has overcome the defects of other alternative approaches, and therefore it is expected to become a potential staple in enhancing thermal stability and usher in a new era of industrial biocatalysis. Nevertheless, several questions have not been illuminated: (1) Whether this method is generally applicable in cyclizing modification of other enzymes? (2) What impact it might exert on the structures and catalytic characteristics of target enzymes?

In the present study, we generated a covalently cyclized lichenase (EC 3.2.1.73, 1,3-1,4-β-glucanase) from *Bacillus subtilis* 168 using SpyTag/SpyCather-mediated cyclization. In detail, we fused lichenase with SpyTag at the N-terminus and SpyCatcher at the C-terminus, and meanwhile ELPs was connected with the C-terminal of SpyCatcher. ELPs are stimulus-responsive polymers constitutive of repeating pentapeptide unit VPGXG, where X represents any amino acid except for proline. Particularly, ELPs can serve as non-chromatographic purification tag that endows recombinant proteins and peptides with substantially elevated purity when undergoing inverse transition cycling (ITC) [23]. This purification method is easy to scale up and can work without resin or any specialized equipment [24]. Here, we use SpyTag/SpyCather-mediated cyclization approach coupled with ITC non-chromatographic purification to obtain the cyclized lichenase with high purity. All data from our report together demonstrated that cyclized lichenase showed a superior thermal tolerance compared with the linear one and may be applied for the biocatalytic conversion of lichenan.

## Results

### Verification for the cyclized conformation of lichenase

**Fig. 1:**
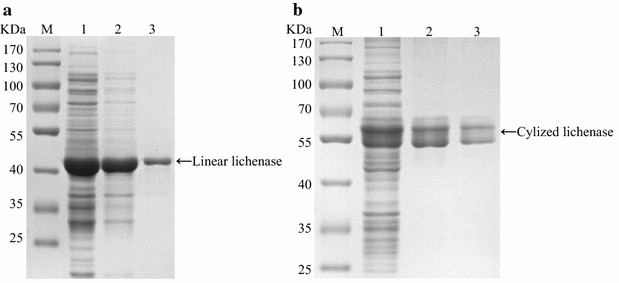
High-expression yield and purity of target fusion proteins were achieved. Products were subjected to SDS-PAGE analysis, before **a** and after **b** the cyclization.* Lanes*: *lane M* marker; *lane 1* the soluble lysates; *lane 2* supernatants after the first-round ITC; *lane 3* supernatants undergoing the second-round ITC

**Fig. 2:**
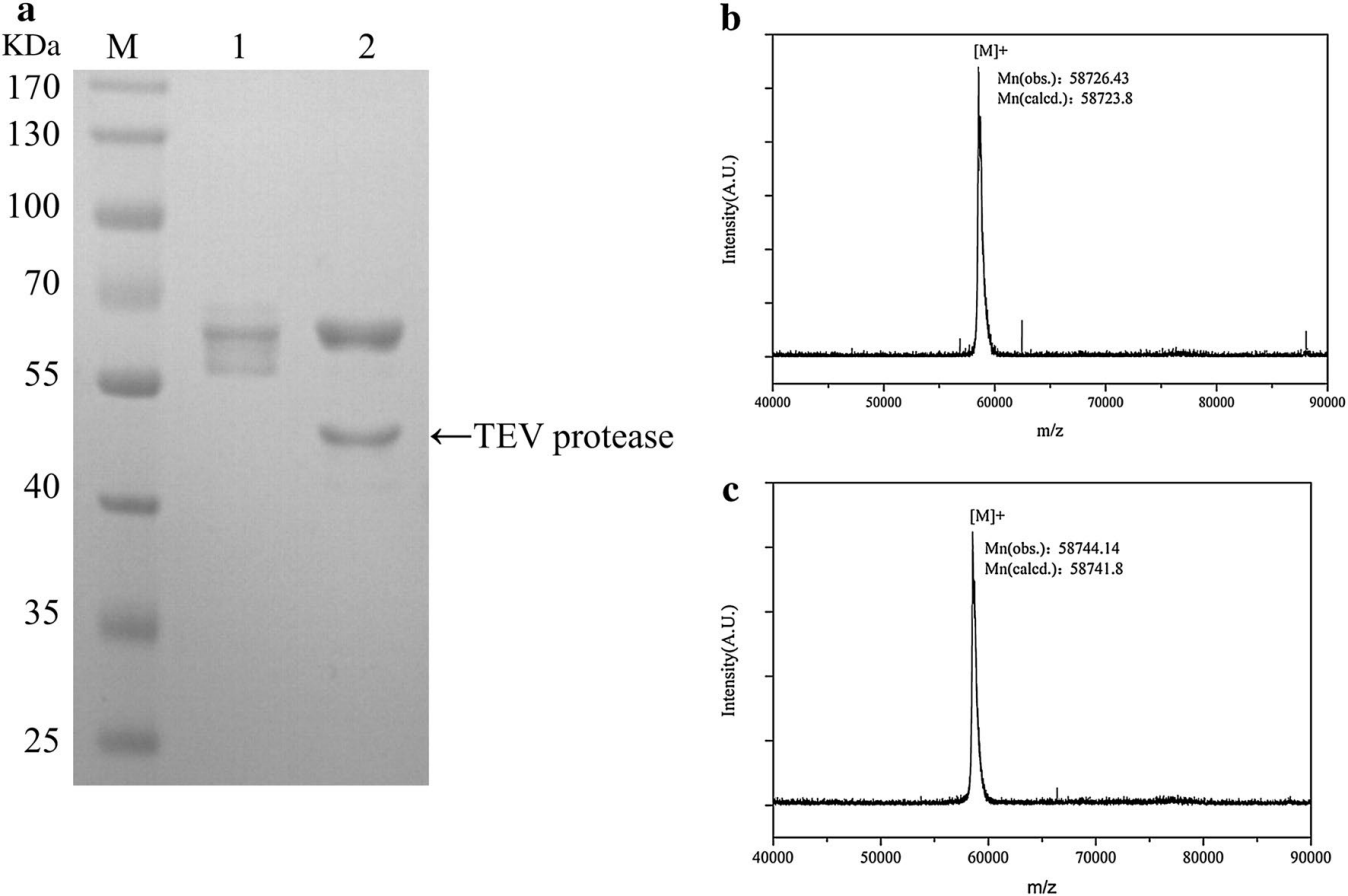
**a** SDS-PAGE analysis of proteolytic digestion products of cyclized lichenase.* Lanes* *lane M* marker; *lane 1* purified cyclized lichenase before digestion; *lane 2* the corresponding products after digestion. The band with an apparent molecular weight of 48 kDa is the TEV protease. **b** and **c** MALDI-TOF mass spectrum of lichenase undergoing cyclization before **b** and after **c** TEV cleavage

The crude lysate was subjected to two-round ITC purification, and the purities of fusion lichenase after each round purification procedure were evaluated by sodium dodecyl sulfate polyacrylamide gel electrophoresis (SDS-PAGE) analysis (Fig. 1). SDS-PAGE yielded two bands of 42 and 58 kDa, denoting the linear and cyclized lichenase with ELPs tag, respectively. Quantity calculating results demonstrated that the linear and cyclized lichenase comprised nearly half (about 50 vs. 40 %, respectively) of the total soluble proteins. Particularly, through the second-round ITC purification, the proportions for linear and cyclized lichenase reached to approximately 97 and 98 %. Besides, the precise molecular weight (MW) of purified cyclized lichenase was further determined by MALDI-TOF mass spectrometer (MS). The MS profiling of the purified product displayed an MW of 58726.43, which matched the theoretical value for cyclized lichenase at 58723.8 calculated by ProtParam tool (Fig. 2b).

To further validate the cyclized topology of lichenase provided by our SpyTag—SpyCatcher covalent reaction, the products were proteolytic digested with tobacco etch virus (TEV) protease and then analyzed by SDS-PAGE and MALDI-TOF MS, as shown in Fig. 2a–c. After such cleavage, only one single band representative of a linear topological enzyme, other than TEV protease, was yielded in the SDS-PAGE. Furthermore, a unique peak at 58744.14 was detected, with a mass 17.71 Da (about 18 Da, the molecular weight of water) less, possibly resulting from loss of water molecule.

### Activity of the cyclized lichenase to linear one

**Fig. 3:**
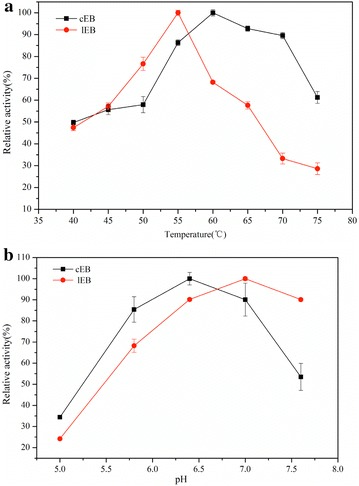
**a** Effect of temperature on the relative activity of linear and cyclized lichenase. **b** Effect of pH on the relative activity of the two forms of lichenase. *cEB* cyclized lichenase, *lEB* linear lichenase. Experiments were performed in triplicate and *error bar* means the standard deviation

To determine the effect of temperature, the activities of the linear and cyclized lichenases were measured at different temperatures, varying from 40 to 75 °C using lichenan as a substrate. As shown in Fig. 3a, the linear and cyclized lichenases exhibited maximum activities at the optimum temperature of 55 and 60 °C, respectively. This indicated that the cyclization process made no dramatic difference on the optimal reaction temperature. However, the cyclized lichenase was operative over broad range of temperature 40–75 °C, whereas the linear one lost majority of its activity when temperature rose to 60 °C. This result showed that the cyclization enhanced the thermal stability of lichenase. Similarly, activities were evaluated over pH range of 5–8. They hold resemblant pH activity behaviors (Fig. 3b) and there was no significant discrepancy between the optimum pH for cyclized lichenase and its linear counterpart (6.4 v.s. 7.0, respectively). This suggested that the cyclized lichenase was acid-tolerant comparable to the linear one, of which the optimal pH was 0.6 units lower.

The lichenase activities before and after the 2nd round ITC were listed in Table 1. The specific activities of the linear and cyclized lichenase were 126.78 and 57.73 (U per mg dry cell weight) after 2nd round ITC, respectively. The specific productivity of the linear and cyclized lichenase were 783.58 and 261.76 U/g dry cell weight with the recovery yield of about 22.18 and 16.95 %, respectively.

**Table 1:** Purification profile of the linear and cyclized lichenase

	Linear lichenase		Cyclized lichenase	
	Before ITC	After ITC	Before ITC	After ITC
Activity (U)	8620.88	1911.94	3768.75	638.70
Protein (mg)	303.98	15.08	339.3	11.05
Specific activity (U/mg)	28.36	126.78	11.10	57.73
specific productivity (U/g)^a^	3533.15	783.58	1544.57	261.76
Activity recovry (%)	22.18		16.95	
Purification fold	4.47		5.20	

The table data were based on 1L culture

^a^ The specific productivity means the activity productivity of dry cell weight (1 L culture)

### Kinetic parameters of the cyclized and linear lichenase

The kinetic parameters were calculated and shown in Table 2. The kinetic profiling of cyclized lichenase was analyzed in comparison with the linear form of lichenase. The Michaelis constant Km value of the cyclized lichenase was approximately 1.7-fold lower than that of the linear one, indicating a higher affinity with specific substrates. The Kcat of cyclized lichenase was much more than double the original linear one, and the Kcat/Km of the cyclized lichenase was about 2.7-fold greater, hinting higher catalytic efficiency.

**Table 2:** Kinetic parameters of the linear and cyclized lichenase

	Vmax (μmol/min/mL)	Km (mg/mL)	Kcat (s^−1^)	Kcat/Km (L/mg/s)
Linear lichenase	4.30 ± 0.47	12.96 ± 1.93	141.14 ± 16.28	10.36 ± 2.43
Cyclized lichenase	2.65 ± 0.16	7.58 ± 0.92	213.2 ± 23.43	28.1 ± 3.44

Experiments were performed in triplicate and error means the standard deviation

### Superior thermal stability of cyclized lichenase to the linear one

**Fig. 4:**
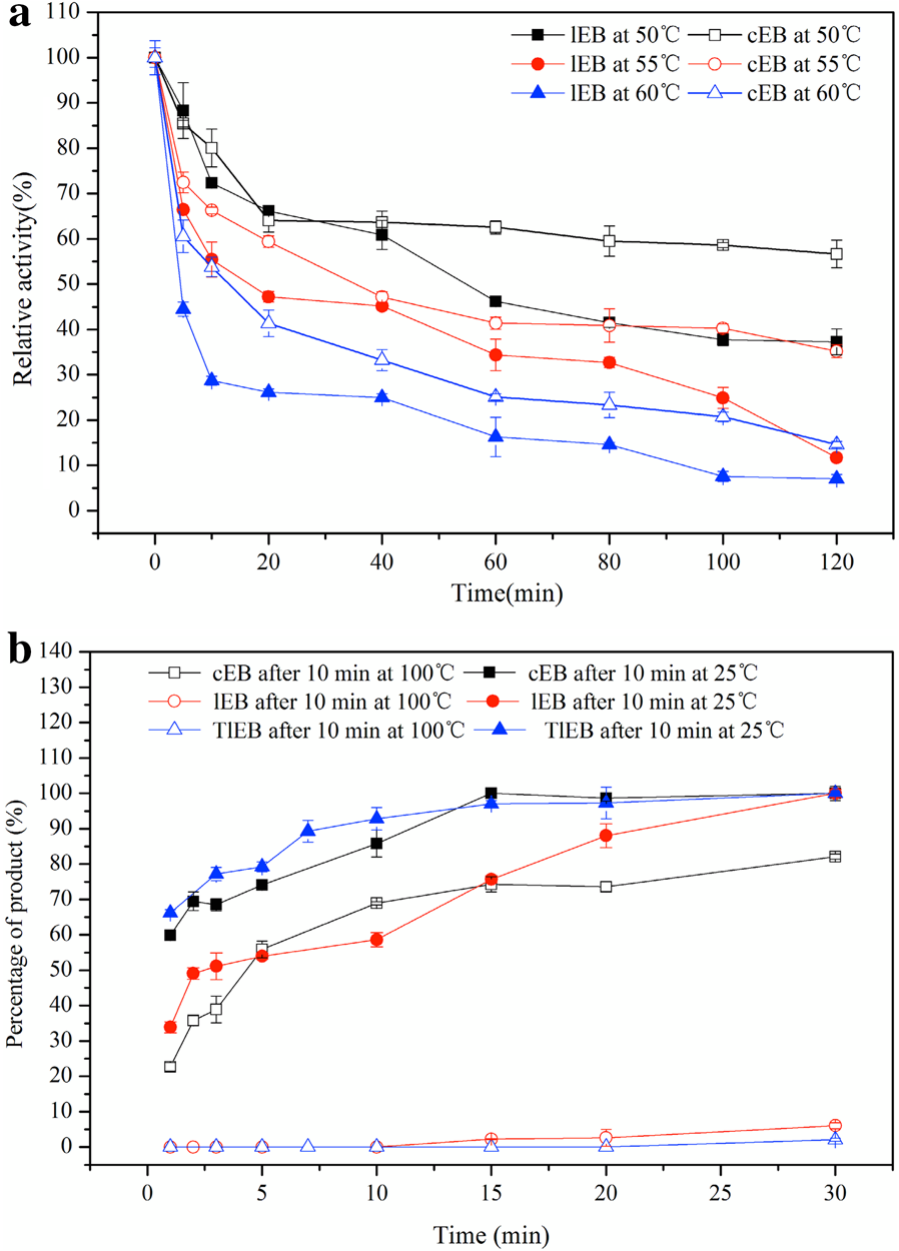
**a** The thermal stability of the linear and cyclized lichenase at various temperature intervals of 50, 55 and 60 °C. **b** Enzyme activity after incubation at room temperature for 10 min or 100 °C heating. Enzyme activity was indicated by the generation rate of product. *cEB* cyclized lichenase, *lEB* linear lichenase, *TlEB* linear topology of product after TEV cleavage following cyclization. Experiments were performed in triplicate and *error bar* means the standard deviation

The thermal stability of the linear lichenase and cyclized lichenase were evaluated based on the residual activities (activity at the outset without heating was defined as 100 %) at various heating periods, as illustrated in Fig. 4a. For all of the three indicated trial temperatures (50, 55, 60 °C), cyclized lichenase show a robuster activity at any of the time intervals tested. Meanwhile, we determined lichenase activities after incubation at room temperature or in boiling water bath for 10 min. As expected, nearly all of the activity of the linear lichenase was irreversible lost upon heating up to 100 °C. On the contrary, more than 80 % of the activity of cyclized lichenase was retained after 100 °C exposure but the catalytic competence of the linear one was barely inactive (Fig. 4b). Given that the linear lichenase carrying SpyTag generated after TVE protease digestion lost almost all of the activity on boiling heating, we can conclude only cyclized topology conferred resilience, and by cyclizing, lichenase could be prevented from denaturation and resist to high temperature. These results unequivocally suggested the lichenase resilience was dramatically enhanced after cyclization.

## Discussions

Inverse transition cycling (ITC) based on the ELPs non-chromatographic tag for recombinant protein purification was originally described by Meyer and Chilkoti in 1999 [25], and has been rapidly developed owing to its merits including versatility, ease to scale up, cost and time efficiencies, and technical simplicity. Several successful trials have been carried out including pilot purifications for β-lactamase, thioredoxin, and xylanase [26, 27]. In this paper, we took lichenase as a model, coupling with ITC method as a tracking and purifying tool, to demonstrate our proposed SpyTag/SpyCather cyclization system in successfully enhancing the stability of lichenase. In addition, high purities at more than 95 % for both linear and cyclized lichenase were visualized through SDS-PAGE analysis.

**Fig. 5:**
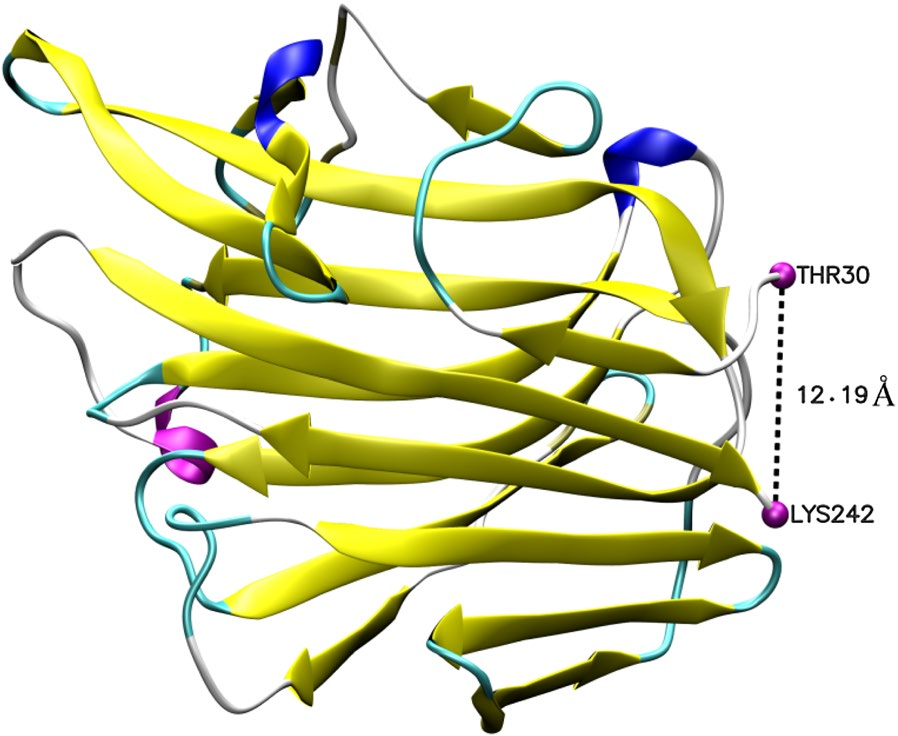
The distance between the N- and C-termini of lichenase. (PDB: 3o5s)

To verify whether the isopeptide bond was formed between the SpyTag and SpyCather, a TEV protease cleavage site was introduced after the BglS. After TEV protease digestion, the products were subjected to MALDI-TOF mass spectrometer (Fig. 2b) and the molecular weight was nearly 18 Da less than the undigested precursor (Fig. 2c), resulting from loss of water molecule. On the other side, twin bands of different molecular weights were observed before TVE cleavage, whereas post-digestion product (linear structure) was represented by one single band signifying that the cyclization efficiency could not reach 100 %. We used the VMD software to calculate the distance between the N- and C-terminal of the lichenase. The 3D model comes from the PDB database (3o5s), as our sequence has 100 % identity with it. The distance between the N- and C-terminal of the lechenase was 12.19 Å as shown in Fig. 5. For single-domain proteins in the PDB, approximately 50 % of them have N- and C-terminal structural elements within 5 Å [28], we supposed the relative long distance might be the reason that caused the incomplete cyclization. In order to further probe into the topological structure of our lichenase, we also performed the activity resilience assay, a manifestation that the activity resilience rate of the cyclized products was 80 % after 100 °C incubation, however, as described by Howarth and coworkers’ study, the cyclized enzyme exhibited approximately 100 % recovery rate after 100 °C exposure [22].

Next, we explored the enzymatic parameters for the cyclized lichenase and compared with its linear counterpart. Cota and colleagues previously reported the optimal pH and temperature of the free lichenase purified by Ni^2+^-chelating affinity and size-exclusion chromatography, and it was 6.4 and 50 °C, respectively. The activity peaks for the cyclized lichenase and the linear one were detected at pH of 6.4 and 7.0, respectively (Fig. 3b), consistent with the study by Cota et al. [29]. The optimum temperature of the cyclized lichenase was 5 °C higher than its linear form (Fig. 3a). Notably, the optimal temperature for cyclized lichenase shifted by 10 °C compared with the free lichenase. Furthermore, we evaluated the thermal stability of both forms of lichenases at various temperatures (Fig. 4a), and meanwhile tested the lichenase activities after incubation at room temperature or 100 °C for 10 min (Fig. 4b). We found that nearly 80 % activity of the cyclized lichenase could be maintained after 100 °C exposure, whereas almost all of the activity of the linear one was denatured upon heating up to 100 °C. This demonstrated the thermal stability was significantly enhanced via the cyclization modification. It was in accordance with the previous study on SpyTag/SpyCather-mediated cyclization by Howarth and coworkers [22]. The author had successfully cyclized β-lactamase as well as dihydrofolate reductase, and verified the SpyTag/SpyCather-mediated cyclization approach could dramatically enhance the protein thermal stability. At present, we have proved this novel cyclization strategy could be expanded to the stabilization of lichenase (a representative model for hydrolytic enzyme). It is suggesting that this cyclization method could potentially serve as a generalized reference for enzyme stability and be customized in various enzymes. On the other hand, cyclized lichenase owned superior substrate affinity and catalytic efficiency than the linear one according to analysis of kinetic panel shown in Table 2. All these results unambiguously certified the cyclization method based on SpyTag/SpyCather reaction could dramatically enhance the lichenase thermal stability, while maintaining its intrinsic structure and biocatalytic activity. This was attributed to the suitable molecular linker we introduced to this process. Moreover, this cyclization method could serve as a general reference for protein stabilization and be customized in various stabilizing requirements based on the characteristics of proteins and peptides. Even for those proteins where N-terminal situated long distance from C-terminal, spontaneous reaction between SpyTag and SpyCather can be fulfilled through judicious design of linkers that were classified into multiple categories (e.g., flexible, rigid, or in vivo cleavable linker) or of different lengths, hence the enzymes cyclized [30].

In addition, the exploitation of the non-chromatographic purification tag ELPs facilitated the purification of cyclized lichenase with higher efficiency and technical simplicity. Based on the purification profile of the linear and cyclized lichenase in Table 1, the specific productivities of the linear and cyclized lichenase were 783.58 and 261.76 U/g with the recovery yields of about 22.18 and 16.95 %, respectively, which was not good enough for industrial application. However, as previously reported by Banki MR et al. [31], the recovery yield of the β-lactamase-ELPs and catalase-ELPs fusion protein were 26.5 and 29.6 %, respectively, which was similar to our study. Meanwhile, according to Mark Shimazu et al. [32], the recovery yield of ELPs–OPH (organophosphorus hydrolase) could reach up to 70 %. Combined with the previous studies by Baley AF and David WW [33], ELPs-tagged proteins can be expressed and purified at high yield using *E. coli* fermentation at high cell density and the yielding could reach 50–120 mg per liter culture in shake flask. Although the recovery yield in our study did not match up with our expectations, the recovery yield could be enhanced by medium and separation process optimization in the follow-up study. Our result also suggested that ELPs fusion did not exert a negative impact on the cyclization efficiency of SpyTag/SpyCather or the lichenase properties including molecular conformation and bioactivity. In our previous study, we covered that ELPs tag and the xylanase fused to ELPs could self-assemble into an insoluble active particle under particular conditions. This not only significantly improved the thermal tolerance, storage stability, and reusability of the enzyme, but this also did not influence the biological catalytic activity [27]. In fact, the relative low recovery rate of this study was partially concerned with the formation of the insoluble active lichenase during the ITC process. We discarded the insoluble active lichenase when purifying the soluble linear and cyclized lichenase as their purities were ranging from 85 to 90 %, which was not enough for theoretical study here, but they may be suitable for practical applications. In this condition, the ELPs fusion tag functioned as a “stabilizer” that was similar to the role carrier plays in the enzyme immobilization. Therefore, it is anticipated that by tuning the reaction parameters, we could achieve the naturally occurring self-assembling between cyclized lichenase and ELPs tag, immediately after the covalent cyclization of the enzyme. Once this was completed, it would not only enable the time- and cost-efficient integration of lichenase immobilization with purification, but also improve the thermal stability and recyclability of the lichenase on the optimum conditions. This certainly prompts further investigation on the production of value-added products or biofuels from the biodegradation of lechenan. From a broader perspective, our method may further expedite the utilization of biological cyclization strategy in the biocatalytic field, as well as providing solid foundations for the industrial application of biocatalysts.

## Conclusions

In summary, the lichenase isolated from *B. subtilis* 168 was successfully cyclized by virtue of the SpyTag/SpyCather-mediated covalent cyclization reaction. The thermal stability of cyclized variant was significantly strengthened to resist the elevated temperatures and facilitate lichenase refolding after thermal stress, overcoming the major defects in biocatalysis such as relatively low thermal stability and limited shelf life. Compared with other conventional chemical cyclization approaches, our strategy exhibits several advantages including technical simplicity, averting the usage of resins or other specialized equipment. Furthermore, the cyclization reaction could spontaneously occur in minutes with high yields. Last but not the least, the isopeptide bond ligation was robust enough to resist heating treatment or competing peptide. Thus, this new cyclization approach exhibits tremendous potential for broad application in the biotechnological and biocatalytic fields.

## Methods

### Plasmid construction

**Fig. 6:**
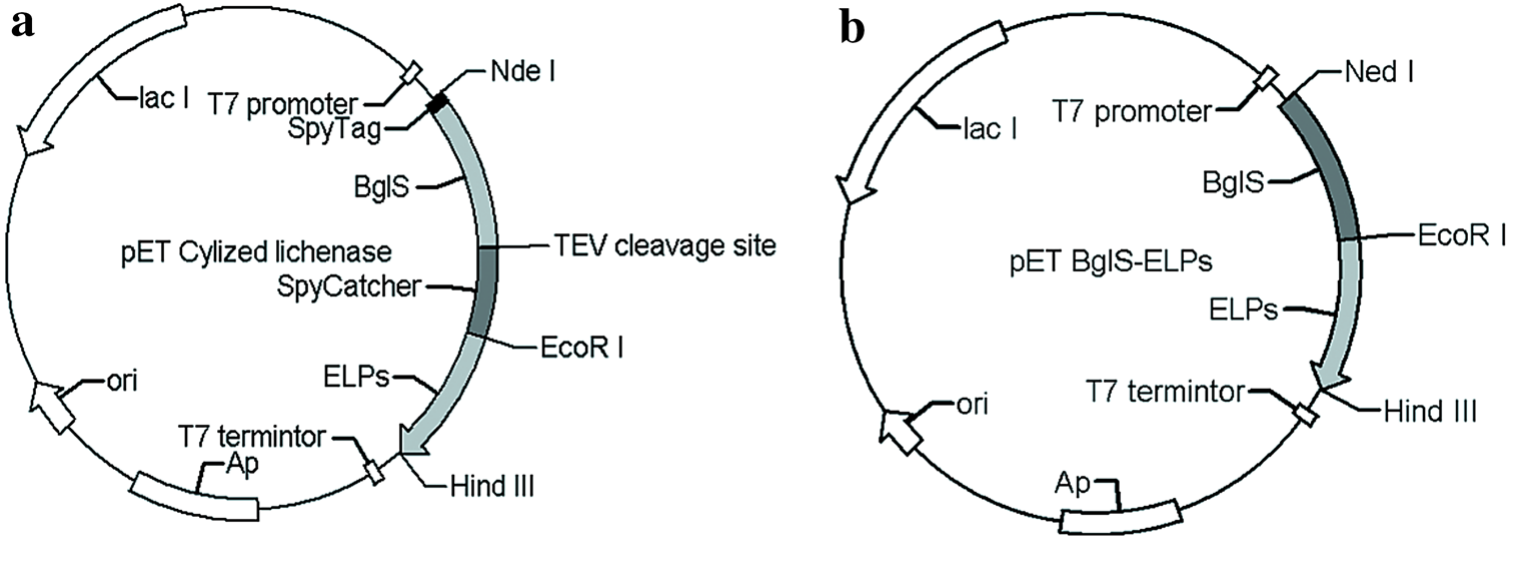
The profiles of plasmid pET-Cylized lichenase (**a**) and pET-linear lichenase (**b**). **a** The TEV cleavage site was inserted between *BglS* gene and *SpyCatcher* gene. The cyclized lichenase gene was cloned between *Ned*I and *Hind*III digestion sites in pET 22(b+), while **b** the linear lichenase fusion gene was cloned into pET 22(b+) using *Ned*I and *Hind*III as restriction enzymes as well

Gene fragments encoding lichenase (*BglS*, Gene ID:937470) [29], SpyTag, and SpyCatcher [34] sequences were synthesized and sequenced by Sangon Biotech Co., Ltd (Shanghai, China) and sequentially cloned into pET 22b(+) vector. A six-residue GSGGSG was used as a linker to connect BglS with *SpyTag* gene. We also inserted a TEV protease cleavage site between BglS and SpyCather in order to confirm that we had successfully cyclized lichenase, meanwhile ELPs was connected with the C-terminal of SpyCatcher (schematically described in Fig. 6a). The gene of the cyclized lichenase was cloned between *Ned*I and *Hind*III digestion sites in pET 22b(+). At the same time, we also constructed a DNA fragment that encodes the linear lichenase, where ELP_40_ directly fused with it, devoid of SpyTag/SpyCather (Fig. 6b). The fusion gene of the linear lichenase was also cloned into pET 22(b+) using *Ned*I and *Hind*III as restriction enzymes as well.

### Protein expression, extraction, and purification

The plasmids carrying the linear lichenase and cyclized lichenase were transformed into *E.coil* strain BL21 (DE3). The stain was cultured overnight at 37 °C in Luria–Bertani (LB) medium containing 100 μg/ml ampicillin and then inoculated into terrific broth (TB) medium for a continued incubation at 37 °C. When the OD_600_ value reached 0.5–0.6, the TB culture were supplemented with 0.5 mM isopropyl-β-thiogalactopyranoside (IPTG) and subjected to an additional incubation at 20 °C for 24 h. *E.coil* cells were harvested by centrifugation at 4 °C (8000×*g*, 15 min). The pellets were resuspended in cold phosphate-buffered saline (PBS, pH 6.4) and homogenized on ice through ultrasonic disruption, followed by centrifugation at 4 °C (16,000×*g*, 10 min) to remove the insoluble matter. The fusion proteins were purified from *E. coli* lysate by using ITC protocol as described elsewhere [23, 35, 36], and by adding the crystalline NaCl (2.5 M) and incubating at 37 °C for 20 min to trigger the ELPs phase transition during the purification step. After that, the sample was centrifuged at 40 °C (16,000×*g*, 10 min) immediately. After discarding the supernatant, the resolubilized pellet was subsequently brought to a final centrifugation at 4 °C (16,000×*g*, 10 min) to remove the insoluble contaminants, followed by the collection of the supernatant containing the lichenases. We performed the ITC procedure twice to improve the purity of the target protein.

### Lichenase activity assay

The enzymatic activities of both the linear and cyclized lichenase were measured using the 3, 5-dinitrosalicylic acid (DNS) method [37]. The standard test was conducted at 55 °C for 10 min in PBS buffer (pH 6.4), using 1.0 % (w/v) lichenan (Megazyme, Wicklow, Ireland) as a substrate and monitored with the OD value at a wavelength of 540 nm. One activity unit was defined as the amount of lichenase that released 1 μmol of reducing sugar per minute. For the optimum temperature determination, a temperature gradient ranging from 40 to 75 °C was carried out. Similarly, pH varied gradually from 5.8 to 7.6 under the optimal temperature of the linear and cyclized lichenase, respectively, for the optimal pH identification. The thermal stabilities for the linear and cyclized lichenase were assessed by incubating lichenase solution for indicated periods (5, 10, 20, 40, 60, 80, 100, 120 min) at the given temperature at 50, 55, and 60 °C, followed by measurement of residual activity under the standard conditions. To evaluate the enzyme resilience, after linear and cyclized lichenase were incubated at 25 °C, or heated for 10 min in a boiling water bath and cooled to room temperature, the activities were assayed by several given reaction time, respectively. The production amounts generated within 30 min were defined as 100 %.

The kinetic parameters were estimated for the linear and cyclized lichenase using substrate concentrations in the range of 1.0–10.0 mg/ml at the optimal temperature and pH in PBS. The Km value and maximum reaction velocity (Vmax) of lichenase were calculated using Lineweaver–Burk plot method.

### Protein characterization

Samples were separated by SDS-PAGE with Coomassie blue staining to determine the protein purity. Matrix-assisted laser desorption ionization mass spectrometry (MALDI-MS) was performed on an UltrafleXtreme MALDI TOF/TOF mass spectrometer (Bruker Daltonics Inc., MA, USA) with sinapinic acid as the matrix, salts were removed from the sample using the ultrafiltration centrifuge tube (Millpore) [34]. The protein concentration was measured by coomassie brilliant blue method using bovine serum albumin serum (BSA) as a reference standard. Proteolytic digestion was carried out using ProTEV Plus (Promega Inc., Madison, USA) based on the manufacturer’s instructions. Typically, 46 μl of protein was added with 2.5 μl of ProTEV buffer (20×), 0.5 μL of 100 mM DTT, and 1 μl of ProTEV Plus. After that, the mixture was incubated overnight at room temperature for sufficient digestion. The molecular weights for corresponding proteins were calculated using ProtParam tool at http://web.expasy.org/protparam/. The 3D model of the lichenase comes from the PDB database (3o5s), as our sequence has 100 % identity with it.

## Abbreviations

ELPs: elastin-like polypeptide
ITC: inverse transition cycling
SDS-PAGE: sodium dodecyl sulfate polyacrylamide gel electrophoresis
MW: molecular weight
MS: mass spectrometer
TEV: tobacco etch virus
LB: Luria–Bertani
TB: terrific broth
IPTG: isopropyl-β-thiogalactopyranoside
PBS: phosphate-buffered saline
DNS: 3, 5-dinitrosalicylic acid
MALDI-MS: matrix-assisted laser desorption ionization mass spectrometry
BSA: bovine serum albumin

## References

[1] Menon V, Divate R, Rao M (2011). Bioethanol production from renewable polymer lichenan using lichenase from an alkalothermophilic *Thermomonospora* sp. and thermotolerant yeast. Fuel Process Technol.

[2] Maktouf S, Moulis C, Miled N, Chaabouni ES, Remaud-Simeon M (2015). A highly thermostable lichenase from *Bacillus* sp. UEB-S: biochemical and molecular characterization. J Mol Catal B: Enzy.

[3] Elgharbi F, Hmida-Sayari A, Sahnoun M, Kammoun R, Jlaeil L, Hassairi H, Bejar S (2013). Purification and biochemical characterization of a novel thermostable lichenase from *Aspergillus niger* US368. Carbohydr Polym.

[4] Tyurin Acapital AC (2015). Sadovskaya NS, Nikiforova Kh R, Mustafaev ON, Komakhin RA, Fadeev VS, Goldenkova-Pavlova IV. *Clostridium thermocellum* thermostable lichenase with circular permutations and modifications in the N-terminal region retains its activity and thermostability. Biochim Biophys Acta.

[5] Polizzi KM, Bommarius AS, Broering JM, Chaparro-Riggers JF (2007). Stability of biocatalysts. Curr Opin Chem Biol.

[6] Bommarius AS, Paye MF (2013). Stabilizing biocatalysts. Chem Soc Rev.

[7] Sun Y, Yang H, Wang W (2011). Improvement of the thermostability and enzymatic activity of cholesterol oxidase by site-directed mutagenesis. Biotechnol Lett.

[8] Yasukawa K, Inouye K (2007). Improving the activity and stability of thermolysin by site-directed mutagenesis. Biochim Biophys Acta.

[9] Lebbink JHG, Kaper T, Bron P, van der Oost J, de Vos WM (2000). Improving low-temperature catalysis in the hyperthermostable *Pyrococcus furiosus* beta-glucosidase CelB by directed evolution. Biochemistry.

[10] Pardo I, Vicente AI, Mate DM, Alcalde M, Camarero S (2012). Development of chimeric laccases by directed evolution. Biotechnol Bioengin.

[11] Denard CA, Ren H, Zhao H (2015). Improving and repurposing biocatalysts via directed evolution. Curr Opin in Chem Biol.

[12] Aboye TL, Camarero JA (2012). Biological synthesis of circular polypeptides. J Biol Chem.

[13] Borra R, Camarero JA (2013). Recombinant expression of backbone-cyclized polypeptides. Biopolymers.

[14] Craik DJ, Cemazar M, Wang CKL, Daly NL (2006). The cyclotide family of circular miniproteins: nature’s combinatorial peptide template. Biopolymers.

[15] Goldenberg DP, Creighton TE (1983). Circular and circularly permuted forms of bovine pancreatic trypsin inhibitor. J Mol Biol.

[16] Williams NK, Liepinsh E, Watt SJ, Prosselkov P, Matthews JM, Attard P, Beck JL, Dixon NE, Otting G (2005). Stabilization of native protein fold by intein-mediated covalent cyclization. J Mol Biol.

[17] Deschuyteneer G, Garcia S, Michiels B, Baudoux B, Degand H, Morsomme P, Soumillion P (2010). Intein-mediated cyclization of randomized peptides in the periplasm of *Escherichia coli* and their extracellular secretion. ACS Chem Biol.

[18] Popp MW, Dougan SK, Chuang TY, Spooner E, Ploegh HL (2011). Sortase-catalyzed transformations that improve the properties of cytokines. Proc Natl Acad Sci U S A/PNAS.

[19] Garcia AE, Tai KP, Puttamadappa SS, Shekhtman A, Ouellette AJ, Camarero JA (2011). Biosynthesis and antimicrobial evaluation of backbone-cyclized alpha-defensins. Biochemistry.

[20] Zakeri B, Howarth M (2010). Spontaneous intermolecular amide bond formation between side chains for irreversible peptide targeting. J Am Chem Soc.

[21] Zakeri B, Fierer JO, Celik E, Chittock EC, Schwarz-Linek U, Moy VT, Howarth M (2012). Peptide tag forming a rapid covalent bond to a protein, through engineering a bacterial adhesin. Proc Natl Acad Sci U S A/PNAS.

[22] Schoene C, Fierer JO, Bennett SP, Howarth M (2014). SpyTag/SpyCatcher cyclization confers resilience to boiling on a mesophilic enzyme. Angew Chem Int Ed.

[23] MacEwan SR, Hassouneh W, Chilkoti A. Non-chromatographic purification of recombinant elastin-like polypeptides and their fusions with peptides and proteins from *Escherichia coli*. J Vis Exp. 2014;(88):e51583. doi:10.3791/51583. 10.3791/51583 PMC4188175 24961229

[24] Hassouneh W, Christensen T, Chilkoti A. Elastin-like polypeptides as a purification tag for recombinant proteins. Curr Protoc Protein Sci. 2010;Chapter 6:Unit 6. 11. 10.1002/0471140864.ps0611s61 PMC3076942 20814933

[25] Meyer DE, Chilkoti A (1999). Purification of recombinant proteins by fusion with thermally-responsive polypeptides. Nat Biotechnol.

[26] Trabbic-Carlson K, Liu L, Kim B, Chilkoti A (2004). Expression and purification of recombinant proteins from *Escherichia coli*: comparison of an elastin-like polypeptide fusion with an oligohistidine fusion. Protein Sci.

[27] Li C, Zhang G (2014). The fusions of elastin-like polypeptides and xylanase self-assembled into insoluble active xylanase particles. J Biotechnol.

[28] Krishna MM, Englander SW (2005). The N-terminal to C-terminal motif in protein folding and function. Proc Natl Acad Sci U S A/PANS.

[29] Cota J, Oliveira LC, Damasio AR, Citadini AP, Hoffmam ZB, Alvarez TM, Codima CA, Leite VB, Pastore G, de Oliveira-Neto M, Murakami MT, Ruller R, Squina FM (2013). Assembling a xylanase-lichenase chimera through all-atom molecular dynamics simulations. Biochim Biophys Acta.

[30] Chen X, Zaro JL, Shen WC (2013). Fusion protein linkers: property, design and functionality. Adv Drug Deliv Rev.

[31] Banki MR, Feng L, Wood DW (2005). Simple bioseparations using self-cleaving elastin-like polypeptide tags. Nat Methods.

[32] Shimazu M, Mulchandani A, Chen W (2003). Thermally triggered purification and immobilization of elastin-OPH fusions. Biotechnol Bioeng.

[33] Fong BA, Wood DW (2010). Expression and purification of ELP-intein-tagged target proteins in high cell density *E. coli* fermentation. Microb Cell Fact.

[34] Zhang WB, Sun F, Tirrell DA, Arnold FH (2013). Controlling macromolecular topology with genetically encoded SpyTag-SpyCatcher chemistry. J Am Chem Soc.

[35] Lim DW, Trabbic-Carlson K, MacKay JA, Chilkoti A (2007). Improved non-chromatographic purification of a recombinant protein by cationic elastin-like polypeptides. Biomacromolecules.

[36] Chow DC, Dreher MR, Trabbic-Carlson K, Chilkoti A (2006). Ultra-high expression of a thermally responsive recombinant fusion protein in *E. coli*. Biotechnol Prog.

[37] Miller GL (1959). Use of dinitrosalicylic acid reagent for determination of reducing sugar. Anal Chem.

